# Optimization of machine tool settings for Spirac hypoid gears by controlling symmetry of contact paths

**DOI:** 10.1038/s41598-024-62488-z

**Published:** 2024-05-21

**Authors:** Jiamin Xuan, Haitao Li, Wei Zhang

**Affiliations:** 1 Cryogenic fluid equipment R&D Zhejiang Engineering Research Center, Zhejiang Institute of Mechanical and Electrical Engineering, Hangzhou, 310053 Zhejiang China; 2 College of Engineering, China Agricultural University, Beijing, 100083 China

**Keywords:** Hypoid gears, Direction angle of contact path, Spirac hypoid gears, Machine tool setting, Face-hobbing, Mechanical engineering, Applied mathematics, Computational methods

## Abstract

A novel optimization method to control the symmetry of contact paths on the concave and convex tooth surfaces of the gear then improves the meshing quality was proposed. By modifying the angular setting of the head cutter when cutting the pinion, the direction angles of the two contact paths are equated to estimate their symmetry. The relation between the direction angles is formulated precisely, the influence of the angular setting on the contact paths is investigated, and the equations for obtaining the values of the machine tool settings are derived. The proposed method is applied to a numerical example of a Spirac hypoid gear pair, and the results reveal that the contact paths on the concave and convex tooth surfaces are approximately symmetrical and the transmission errors of both sides are comparable.

## Introduction

Hypoid gears are widely used to transfer power between two non-intersecting crossed axes, mostly found in the front and the rear axles of all-wheel-drive vehicles or in the rear axles of rear-wheel-drive ones^1,3^. Furthermore, most hypoid gears are manufactured by either the face-milling method or the face-hobbing method^2–4^. The face-milling method mainly depends on local syntheses^5–8^, which predetermine the contact characteristics of the pinion and the gear at a mean point, including the direction of the contact path. Besides determining the contact characteristics around the mean point, Wu et al.^9^ presented a theory for the function-oriented design of point contact tooth surfaces. The theory was applied to determine the contact path, major axial length of the instantaneous contact ellipse, and higher-order acceleration as required. Compared with the face-milling method, the face-hobbing method only considers the first-order parameters at the mean point in computing the machine tool settings, such as the position of the reference point, pressure angle, and spiral angle at the reference point; nevertheless, it does not consider the contact path^10^. To guarantee the quality of hypoid gears, various strategies, including higher-order modifications to correct the tooth flank form machining errors^11,12^, ease-off flank modifications for tooth correction and meshing performance improvement^13–15^, and multi-objective tooth optimization with modification-based loaded tooth contact analysis to improve the overall meshing quality^16,17^ have been proposed. However, most of them are based on Gleason’s hypoid generator.

There are three common face-hobbing systems for computing the machine tool settings for hypoid gears, including Klingelnberg’s CycloPalloid system, Oerlikon’s Spirac and Spiroflex systems, and Gleason’s Phoenix system^4,18^. In the Spiroflex system, the pinion and the gear are cut using the generated method; the contact paths on the concave and convex tooth surfaces of the gear are approximately symmetrical, and the instantaneous contact ellipses at the reference point are also symmetrical. However, in the Spirac system, the pinion is cut by the generated method, the gear is cut by the nongenerated method (which enhances production), and the symmetry of the contact paths cannot be guaranteed. Consequently, the contact paths must be modified artificially and repeatedly by correcting the machine tool settings during the tooth contact analysis. The Spirac system is applicable under two conditions for the hypoid gears: when the transmission ratio is greater than or equal to 3 and when the pitch angle of the gear is greater than or equal to 60°^10^. Generally, the hypoid gears cut by the Spirac system are called Spirac hypoid gears. For Spirac hypoid gears, few studies have been conducted to develop a universal hypoid generator mathematical model^19^, a flank-correction methodology from the six-axis Cartesian-type CNC hypoid generator^18^, anew analytical method for the basic machine tool settings to realize conjugated action^20^, an active tooth surface–design methodology based on coordinate measurements^21^, etc.

The contact path is one of the dominant factors governing the load behaviors of hypoid gears ^10^. In this paper, a new method is proposed to calculate the machine tool settings for Spirac hypoid gears. It controls the direction angles of the contact paths to be equated to achieve symmetrical contact paths on both tooth surfaces of the gear. Furthermore, the relation between the direction angles is formulated, the influence of the angular setting of the pinion head cutter on the contact paths is determined, and the equations to solve for the values of the machine tool settings are derived. The computation is tested using a numerical example.

## Theoretical background

**Figure 1:**
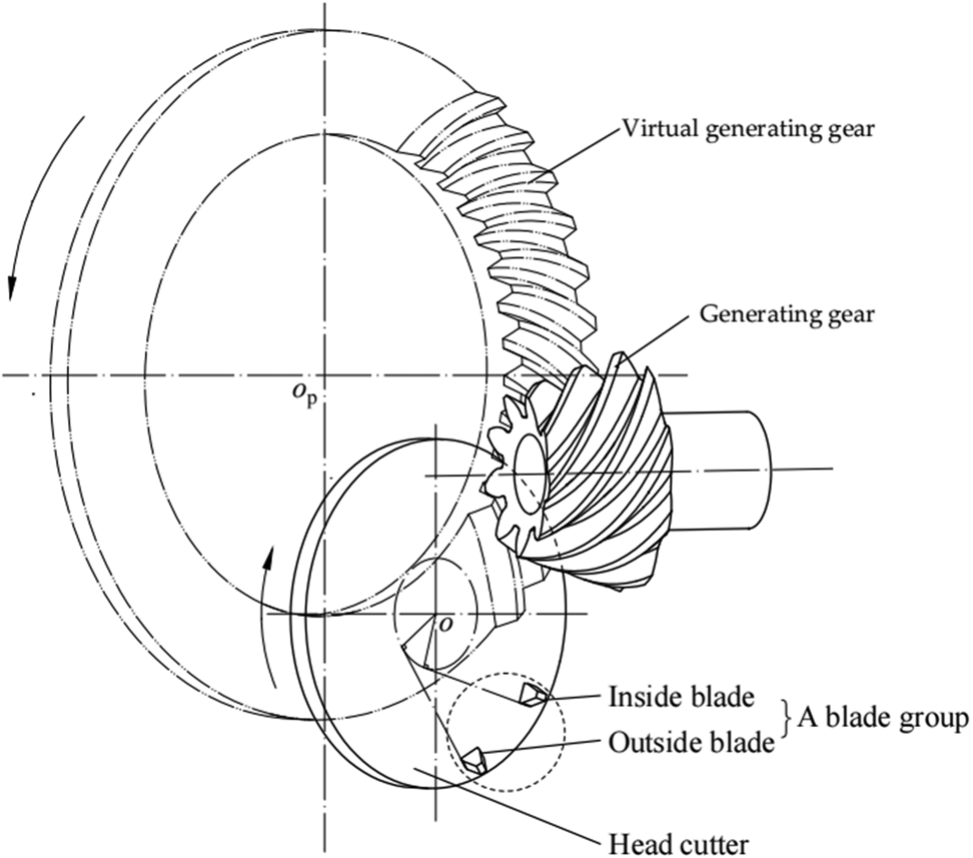
Face-hobbing method.

In the face-hobbing method, the cutting of tooth surfaces is a continuous indexing process. As shown in Fig. 1, the concave and convex tooth surfaces of a tooth slot are manufactured simultaneously using blade groups^12^. Each blade group contains an inside blade and an outside one, which are placed at the same reference circle on the pitch plane of the head cutter. In the face-hobbing method, the traces of the cutting edges of the head cutter blades form the teeth of virtual generating gear. The curve of the tooth trace of the generating gear forms an extended epicycloid.

In the generated method, two sets of related motions are defined: the relative rotation between the head cutter and the blank, and the rolling (or generating) motion, corresponding to the relative rotation between the virtual generating gear and the blank. In the nongenerated method, which is usually applied to the gear, only the indexing motion is considered.

### Generation of the gear tooth surface

**Figure 2:**
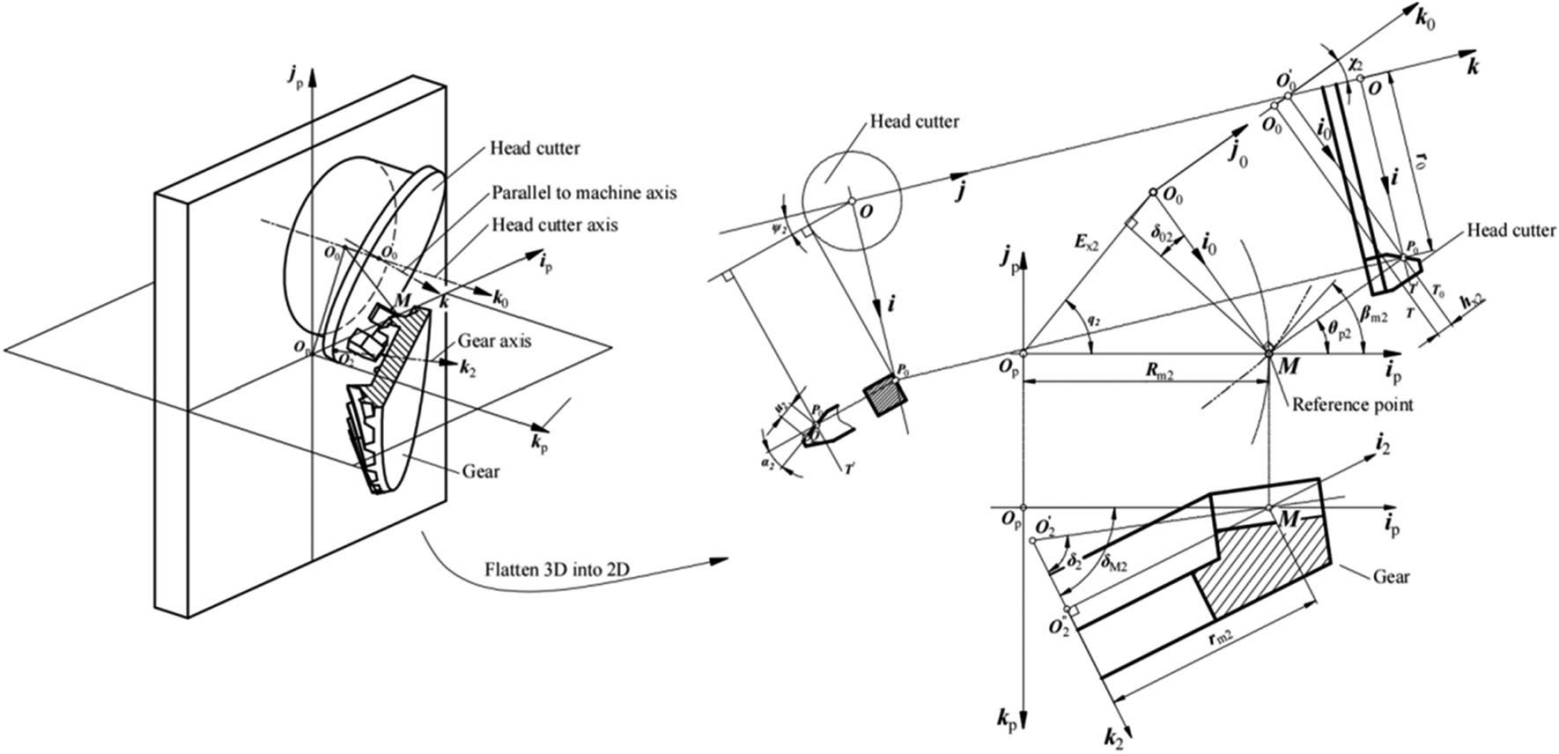
Relative position between the head cutter and the gear with the right-hand teeth.

When the gear is cut by the nongenerated method, the trace of the blade on the blank is the tooth surface of the gear. Figure 2 shows the relative position between the head cutter and the gear with the right-hand teeth. The reference plane, *T*, is the basis of the relative position between the gear and the generating gear. *P*_0_ is the pitch point of the blades and its distance to the axis of the head cutter is the nominal cutter radius (*r*_0_). *T*_0_ is a plane parallel to plane *T* and that passes through point *P*_0_ with the addendum modification (*h*_x2_). *T*′ is a plane perpendicular to the axis of the head cutter and rotating about point *P*_0_ by tilt angle of the head cutter (*χ*_2_). *M* is the reference point of either the pinion or the gear. In order to accommodate the correct orientation with respect to the cutting motion vector, the effective cutting direction of the blades in the head cutter is not perpendicular to the cutter radius vector. *δ*_02_ is the angle between the cutting direction of the blade and the cutter radius vector. *β*_m2_ is the mean spiral angle of the gear at point *M*. *R*_m2_ is the gear cone distance of point *M*. *r*_m2_ is the radius of the reference circle at point *M*. *δ*_M2_ is the gear installment angle. When angle *δ*_M2_ equals the gear pitch angle *δ*_2_, origin *O*_p_ coincides with the gear pitch cone apex *O*′_2_.

Coordinate system *S*(*O*; ***i***, ***j***, ***k***) is rigidly connected to the head cutter, where origin *O* is at the intersection point of plane *T*′ and the axis of the head cutter, axis ***i*** directs to point *P*_0_, and axis ***k*** coincides with the axis of the head cutter. Coordinate system *S*_0_(*O*’_0_; ***i***_0_, ***j***_0_, ***k***_0_) is an auxiliary coordinate system, where origin *O*’_0_ is at the intersection point of plane *T*_0_ and the axis of the head cutter, axis ***i***_0_ directs to point *P*_0_, and axis ***k***_0_ is perpendicular to plane *T*_0_. Origin *O*_0_ is at the intersection point of plane *T* and axis ***k***_0_, initially located based on the radial setting (*E*_x2_) and the angular setting (*q*_2_) in coordinate system *S*_p_(*O*_p_; ***i***_p_, ***j***_p_, ***k***_p_). Axis ***i***_p_ passes through point *M*, and axis ***k***_p_ coincides with axis ***k***_0_. Coordinate system *S*_2_(*O*″_2_;***i***_2_,*** j***_2_, ***k***_2_) is rigidly connected to the gear, where origin *O*″_2_ is at the center of the reference circle of the gear; axis ***i***_2_ passes through point *M*, axis ***j***_2_ coincides with axis ***j***_p_, and axis ***k***_2_ coincides with the axis of the gear and points to the heel.

The trace of the cutting edge of the head cutter blade is presented in coordinate system *S* (rigidly connected to the head cutter) by the vector-parametric equation (fully described in Reference^22^):

1 $$\left({{\varvec{R}}}_{2}\right)={\varvec{O}}{{\varvec{P}}}_{0}+{{\varvec{P}}}_{0}{\varvec{Q}}={{\varvec{R}}}_{2}\left({u}_{2},{\psi }_{2},{\alpha }_{2}\right),$$

Where (***R***_2_) is a position vector of an arbitrary point of the trace of the cutting edge; *u*_2_ is the distance from the intersection point of the edge of the blade and plane *T*′ to an arbitrary point along the edge of the blade; *ψ*_2_ is the angle of rotation of the head cutter; *α*_2_ is the blade angle. The subscript inside the parentheses indicates the number of a body the considered quantity belongs to (index 1 indicates a pinion, index 2 indicates a gear, index 4 indicates a generating gear of pinion). The subscript outside the parentheses indicates a coordinate system in which the considered vector is defined.

The gear tooth surface (*Σ*_2_) produced by coordinate transformation from coordinate system *S* to coordinate system *S*_2_ (connected to the gear) is defined by the following equation (based on Fig. 2)^22^:

2 $$({{\varvec{r}}}_{2}^{*}{)}_{2}={{\varvec{M}}}_{22}\cdot {{\varvec{M}}}_{P2}\cdot {{\varvec{M}}}_{20P}\cdot {{\varvec{M}}}_{20}\cdot \left({{\varvec{R}}}_{2}\right),$$

The coordinate transformations between coordinate systems *S*, *S*_0_, *S*_p_, *S*_2_ (Fig. 2), are performed as it follows:

3 $$({{\varvec{R}}}_{2}{)}_{0}={{\varvec{M}}}_{20}\cdot ({{\varvec{R}}}_{2})=\left[\begin{array}{cc}\begin{array}{c}\mathit{cos}{\chi }_{2}\\ 0\\ \begin{array}{c}-\mathit{sin}{\chi }_{2}\\ 0\end{array}\end{array}& \begin{array}{ccc}0& \mathit{sin}{\chi }_{2}& 0\\ 1& 0& 0\\ \begin{array}{c}0\\ 0\end{array}& \begin{array}{c}\mathit{cos}{\chi }_{2}\\ 0\end{array}& \begin{array}{c}{r}_{0}\cdot \mathit{tan}{\chi }_{2}\\ 1\end{array}\end{array}\end{array}\right]\cdot ({{\varvec{R}}}_{2}),$$

4 $$({{\varvec{r}}}_{2}{)}_{p}={{\varvec{M}}}_{20P}\cdot ({{\varvec{R}}}_{2}{)}_{0}=\left[\begin{array}{cc}\begin{array}{c}\mathit{cos}\left({\theta }_{p2}-{90}^{^\circ }\right)\\ \mathit{sin}\left({\theta }_{p2}-{90}^{^\circ }\right)\\ \begin{array}{c}0\\ 0\end{array}\end{array}& \begin{array}{ccc}-\mathit{sin}\left({\theta }_{p2}-{90}^{^\circ }\right)& 0& {E}_{x2}\cdot \mathit{cos}{q}_{2}\\ \mathit{cos}\left({\theta }_{p2}-{90}^{^\circ }\right)& 0& {E}_{x2}\cdot \mathit{sin}{q}_{2}\\ \begin{array}{c}0\\ 0\end{array}& \begin{array}{c}1\\ 0\end{array}& \begin{array}{c}{h}_{x2}\\ 1\end{array}\end{array}\end{array}\right]\cdot ({{\varvec{R}}}_{2}{)}_{0},$$

5 $$({{\varvec{r}}}_{2}{)}_{2}={{\varvec{M}}}_{P2}\cdot ({{\varvec{r}}}_{2}{)}_{p}=\left[\begin{array}{cc}\begin{array}{c}\mathit{sin}{\delta }_{M2}\\ 0\\ \begin{array}{c}\mathit{cos}{\delta }_{M2}\\ 0\end{array}\end{array}& \begin{array}{ccc}0& -\mathit{cos}{\delta }_{M2}& {R}_{m2}-{r}_{m2}\cdot \mathit{sin}{\delta }_{M2}\\ 1& 0& 0\\ \begin{array}{c}0\\ 0\end{array}& \begin{array}{c}\mathit{sin}{\delta }_{M2}\\ 0\end{array}& \begin{array}{c}{r}_{m2}\cdot \mathit{cos}{\delta }_{M2}\\ 1\end{array}\end{array}\end{array}\right]\cdot ({{\varvec{r}}}_{2}{)}_{p},$$

Where

6 $${\theta }_{p2}={\beta }_{m2}-{\delta }_{02}.$$

To obtain surface *Σ*_2_ in the generating process, the head cutter is rolled with the work gear, and surface *Σ*_2_ are defined by the following:

7 $$({{\varvec{r}}}_{2}^{*}{)}_{2}={{\varvec{M}}}_{22}\cdot ({{\varvec{r}}}_{2}{)}_{2}=\left[\begin{array}{cc}\begin{array}{c}\mathit{cos}\left({i}_{20}\cdot {\psi }_{2}\right)\\ \mathit{sin}\left({i}_{20}\cdot {\psi }_{2}\right)\\ \begin{array}{c}0\\ 0\end{array}\end{array}& \begin{array}{ccc}-\mathit{sin}\left({i}_{20}\cdot {\psi }_{2}\right)& 0& 0\\ \mathit{cos}\left({i}_{20}\cdot {\psi }_{2}\right)& 0& 0\\ \begin{array}{c}0\\ 0\end{array}& \begin{array}{c}1\\ 0\end{array}& \begin{array}{c}0\\ 1\end{array}\end{array}\end{array}\right]\cdot ({{\varvec{r}}}_{2}{)}_{2}=[{x}_{2}{y}_{2}{z}_{2}1{]}^{T},$$

Where the velocity ratio *i*_20_ in the kinematic scheme of the head cutter for the generation of gear tooth surfaces, based on the ratio of the numbers of blades (*z*_0_) and teeth (*z*_2_), is:

8 $${i}_{20}=\frac{{z}_{0}}{{z}_{2}}.$$

The unit normal vector to surface *Σ*_2_ at point *M* in coordinate system *S*_2_ is:

9 $$({{\varvec{n}}}_{2}^{*}{)}_{2}=\frac{{\left({{\varvec{r}}}_{{2}{\text{u}}}^{*}\right)}_{2}\times {\left({{\varvec{r}}}_{{2}\psi }^{*}\right)}_{2}}{\left|{\left({{\varvec{r}}}_{{2}{\text{u}}}^{*}\right)}_{2}\times {\left({{\varvec{r}}}_{{2}\psi }^{*}\right)}_{2}\right|}=\frac{\frac{\partial ({{\varvec{r}}}_{2}^{*}{)}_{2}}{\partial {u}_{2}}\times \frac{\partial ({{\varvec{r}}}_{2}^{*}{)}_{2}}{\partial {\psi }_{2}}}{\left|\frac{\partial ({{\varvec{r}}}_{2}^{*}{)}_{2}}{\partial {u}_{2}}\times \frac{\partial ({{\varvec{r}}}_{2}^{*}{)}_{2}}{\partial {\psi }_{2}}\right|}$$

#### Generation of the generating gear tooth surface of pinion

**Figure 3:**
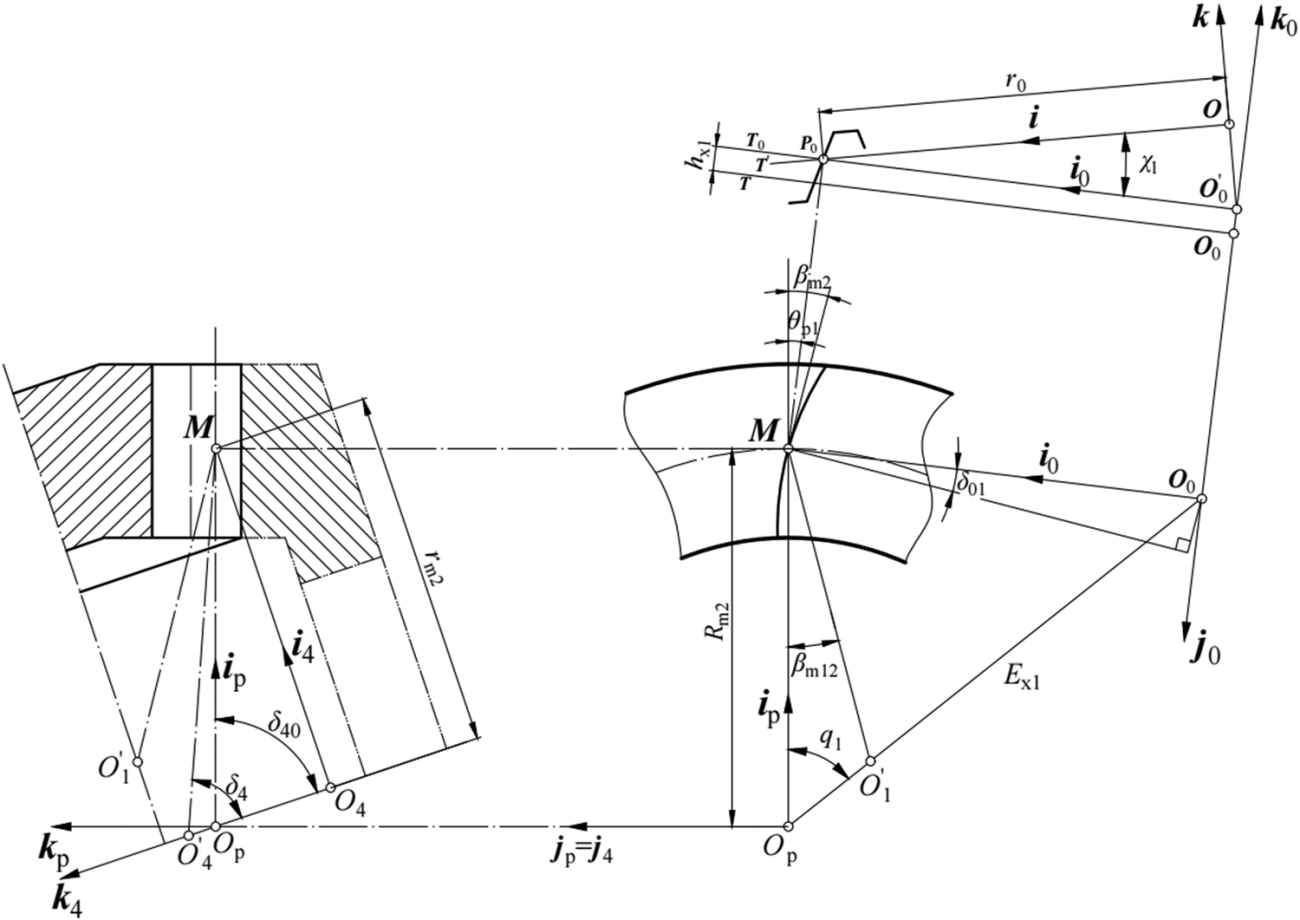
Positions of the head cutter, generating gear, and pinion with the left-hand teeth.

In the generated method, the generation of the pinion is based on the concept of the virtual generating gear, and the pinion tooth surface (*Σ*_1_) is generated as an envelope of the virtual generating gear (*Σ*_4_). Figure 3 shows the relative position between the head cutter, generating gear of pinion, and pinion with the left-hand teeth. The virtual generating gear is similar to the gear. *h*_x1_ is the addendum modification, and *χ*_1_ is the tilt angle of the head cutter. Coordinate systems *S* and *S*_0_ are established similar to the coordinate systems of the gear shown in Fig. 2. Coordinate system *S*_4_(*O*_4_; ***i***_4_, ***j***_4_, ***k***_4_) is connected to the virtual generating gear, where origin *O*_4_ is at the center of the pitch circle of the generating gear, axis ***i***_4_ passes through point *M*, axis ***j***_4_ coincides with axis ***j***_p_, and axis ***k***_4_ coincides with the axis of the generating gear of pinion. Origin *O*_0_ is at the intersection point of plane *T* and axis ***k***_0_, initially located based on the radial setting (*E*_x1_) and the angular setting (*q*_1_) in coordinate system *S*_p_(*O*_p_; ***i***_p_, ***j***_p_, ***k***_p_). *δ*_4_ is the pitch angle of the generating gear. *δ*_40_ is the generating gear installment angle.

The movable coordinate system *S*_4_ is rigidly connected with the generating gear of pinion. It is constructed by analogy to system *S*_4_. The generating gear tooth surface of pinion (*Σ*_4_) and the unit normal to surface *Σ*_4_ at point *M* are ^22^

10 $$({{\varvec{r}}}_{4}^{*}{)}_{4}={{\varvec{M}}}_{44}\cdot {{\varvec{M}}}_{P4}\cdot {{\varvec{M}}}_{40P}\cdot {{\varvec{M}}}_{40}\cdot ({{\varvec{R}}}_{4})=[{x}_{4}{y}_{4}{z}_{4} 1{]}^{T},$$

11 $$({{\varvec{n}}}_{4}^{*}{)}_{4}=\frac{{\left({{\varvec{r}}}_{{4}{\text{u}}}^{*}\right)}_{4}\times {\left({{\varvec{r}}}_{{4}\psi }^{*}\right)}_{4}}{\left|{\left({{\varvec{r}}}_{{4}{\text{u}}}^{*}\right)}_{4}\times {\left({{\varvec{r}}}_{{4}\psi }^{*}\right)}_{4}\right|}=\frac{\frac{\partial ({{\varvec{r}}}_{4}^{*}{)}_{4}}{\partial {u}_{1}}\times \frac{\partial ({{\varvec{r}}}_{4}^{*}{)}_{4}}{\partial {\psi }_{1}}}{\left|\frac{\partial ({{\varvec{r}}}_{4}^{*}{)}_{4}}{\partial {u}_{1}}\times \frac{\partial ({{\varvec{r}}}_{4}^{*}{)}_{4}}{\partial {\psi }_{1}}\right|}.$$

Here, (***R***_4_) is a position vector of an arbitrary point of the trace of the cutting edge, presented in system *S* by the vector-parametric equation (fully described in Reference 22):

12 $$({{\varvec{R}}}_{4})={{\varvec{R}}}_{4}({u}_{1},{\psi }_{1},{\alpha }_{1}),$$

Where *u*_1_ is the distance from the intersection point of the edge of the blade and plane *T*′ to an arbitrary point along the edge of the blade, *ψ*_1_ is the angle of rotation of the head cutter, and *α*_1_ is the blade angle. Subscript 4 inside the parentheses indicates the parameter of the generating gear of the pinion. *x*_4_, *y*_4_, and *z*_4_ are Cartesian coordinates representing the position of (r^*^_4_)_4_.

Matrices ***M***_*i*_ provide the coordinate transformations from coordinate systems *S* to *S*_4_, defined by equations:

13 $$({{\varvec{R}}}_{4}{)}_{0}={{\varvec{M}}}_{40}\cdot ({{\varvec{R}}}_{4})=\left[\begin{array}{cc}\begin{array}{c}\mathit{cos}{\chi }_{1}\\ 0\\ \begin{array}{c}-\mathit{sin}{\chi }_{1}\\ 0\end{array}\end{array}& \begin{array}{ccc}0& \mathit{sin}{\chi }_{1}& 0\\ 1& 0& 0\\ \begin{array}{c}0\\ 0\end{array}& \begin{array}{c}\mathit{cos}{\chi }_{1}\\ 0\end{array}& \begin{array}{c}{r}_{0}\cdot \mathit{tan}{\chi }_{1}\\ 1\end{array}\end{array}\end{array}\right]\cdot ({{\varvec{R}}}_{4}),$$

14 $$({{\varvec{r}}}_{4}{)}_{p}={{\varvec{M}}}_{40P}\cdot ({{\varvec{R}}}_{4}{)}_{0}=\left[\begin{array}{cc}\begin{array}{c}\mathit{cos}\left({\theta }_{p1}-{90}^{^\circ }\right)\\ \mathit{sin}\left({\theta }_{p1}-{90}^{^\circ }\right)\\ \begin{array}{c}0\\ 0\end{array}\end{array}& \begin{array}{ccc}-\mathit{sin}\left({\theta }_{p1}-{90}^{^\circ }\right)& 0& {E}_{x1}\cdot \mathit{cos}{q}_{1}\\ \mathit{cos}\left({\theta }_{p1}-{90}^{^\circ }\right)& 0& {E}_{x1}\cdot \mathit{sin}{q}_{1}\\ \begin{array}{c}0\\ 0\end{array}& \begin{array}{c}1\\ 0\end{array}& \begin{array}{c}{h}_{x1}\\ 1\end{array}\end{array}\end{array}\right]\cdot ({{\varvec{R}}}_{4}{)}_{0},$$

15 $$({{\varvec{r}}}_{4}{)}_{4}={{\varvec{M}}}_{P4}\cdot ({{\varvec{r}}}_{4}{)}_{p}=\left[\begin{array}{cc}\begin{array}{c}\mathit{sin}{\delta }_{40}\\ 0\\ \begin{array}{c}-\mathit{cos}{\delta }_{40}\\ 0\end{array}\end{array}& \begin{array}{ccc}0& \mathit{cos}{\delta }_{40}& {R}_{m2}-{r}_{m2}\cdot \mathit{sin}{\delta }_{40}\\ 1& 0& 0\\ \begin{array}{c}0\\ 0\end{array}& \begin{array}{c}\mathit{sin}{\delta }_{40}\\ 0\end{array}& \begin{array}{c}{-r}_{m2}\cdot \mathit{cos}{\delta }_{40}\\ 1\end{array}\end{array}\end{array}\right]\cdot ({{\varvec{r}}}_{4}{)}_{p},$$

Where

16 $${\theta }_{p1}={\beta }_{m1}-{\delta }_{01},$$

Matrix ***M***_44_ defines the relation between the pinion and its generating gear rotating through mesh, and it is described by the following equation (Fig. 3):

17 $$({{\varvec{r}}}_{4}^{*}{)}_{4}={{\varvec{M}}}_{44}\cdot ({{\varvec{r}}}_{4}{)}_{4}=\left[\begin{array}{cc}\begin{array}{c}\mathit{cos}\left({i}_{20}\cdot {\psi }_{1}\right)\\ \mathit{sin}\left({i}_{20}\cdot {\psi }_{1}\right)\\ \begin{array}{c}0\\ 0\end{array}\end{array}& \begin{array}{ccc}-\mathit{sin}\left({i}_{20}\cdot {\psi }_{1}\right)& 0& 0\\ \mathit{cos}\left({i}_{20}\cdot {\psi }_{1}\right)& 0& 0\\ \begin{array}{c}0\\ 0\end{array}& \begin{array}{c}1\\ 0\end{array}& \begin{array}{c}0\\ 1\end{array}\end{array}\end{array}\right]\cdot ({{\varvec{r}}}_{4}{)}_{4}.$$

### Curvature parameters of the tooth surfaces in the tooth trace and tooth profile directions at the reference point

**Figure 4:**
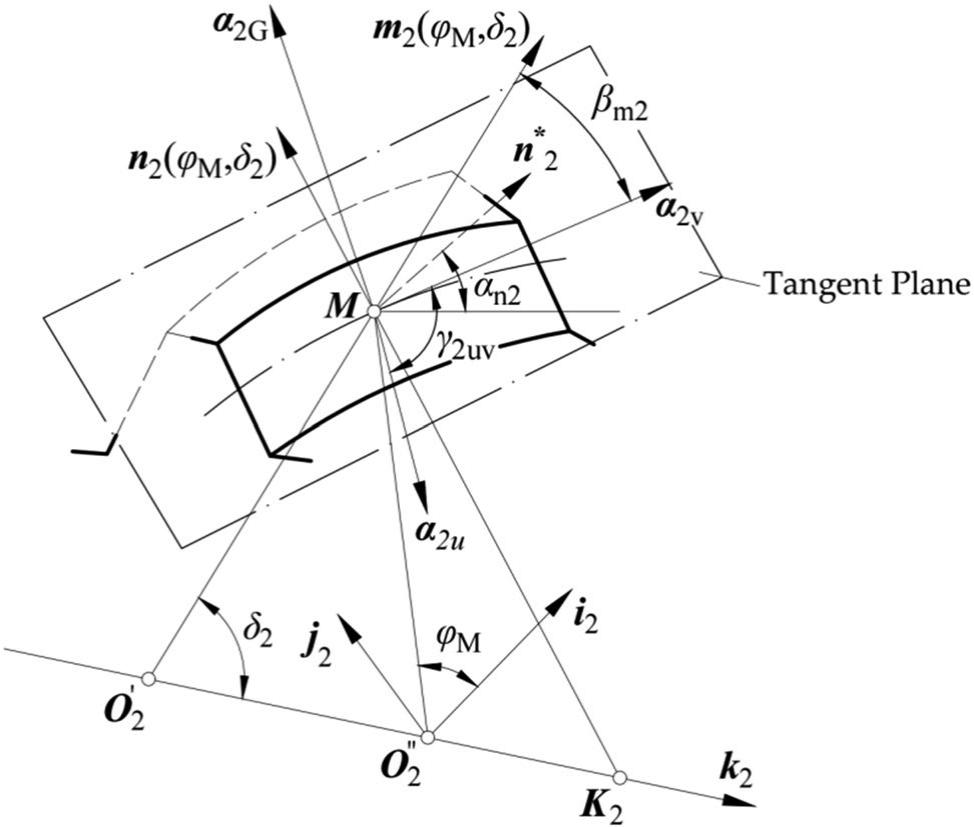
Positions of the tangent vectors to the parameter curves, tooth profile curve, and toothtrace curve at point *M*.

Each of the tooth surfaces described above covers two families of parameter curves, namely, the *u*-parameter curve and the *ψ*-parameter curve. For each point on surface *Σ*_2_, tangent vector ***α***_2u_ to the *u*-parameter curve and tangent vector ***α***_2*ψ*_ to the *ψ*-parameter curve are represented in coordinate system *S*_2_, as follows (based on Fig. 4):

18 $${\left({\boldsymbol{\alpha }}_{\text{2u}}\right)}_{2}=\frac{{\left({{\varvec{r}}}_{{2}{\text{u}}}^{*}\right)}_{2}}{\left|{\left({{\varvec{r}}}_{{2}{\text{u}}}^{*}\right)}_{2}\right|},$$

19 $${\left({\boldsymbol{\alpha }}_{2\psi }\right)}_{2}=\frac{{\left({{\varvec{r}}}_{{2}\psi }^{*}\right)}_{2}}{\left|{\left({{\varvec{r}}}_{{2}\psi }^{*}\right)}_{2}\right|}.$$

The angle between vector ***α***_2u_ and vector ***α***_2ψ_ is determined by

20 $${\gamma }_{2u\psi }=\mathit{arccos}\left[({\boldsymbol{\alpha }}_{\text{2u}}{)}_{2}\cdot ({\boldsymbol{\alpha }}_{2\psi }{)}_{2}\right].$$

The normal curvature and the geodesic torsion of surface *Σ*_2_ for the directions of the *u*-parameter curve and the *ψ*-parameter curve are determined and denoted by *κ*_2*u*_, *τ*_2*u*_, *κ*_2*ψ*_ and *τ*_2*ψ*_, respectively:

21 $${\kappa }_{2u}=0,$$

22 $${\kappa }_{2\psi }=\frac{({{\varvec{n}}}_{2}^{*}{)}_{2}\cdot {\left({{\varvec{r}}}_{{2}\psi \psi }^{*}\right)}_{2}}{{\left[{\left({{\varvec{r}}}_{{2}\psi }^{*}\right)}_{2}\right]}^{2}}=\frac{({{\varvec{n}}}_{2}^{*}{)}_{2}\cdot \frac{{\partial }^{2}({{\varvec{r}}}_{2}^{*}{)}_{2}}{{\left(\partial {\psi }_{2}\right)}^{2}}}{{\left[\frac{\partial ({{\varvec{r}}}_{2}^{*}{)}_{2}}{\partial {\psi }_{2}}\right]}^{2}},$$

23 $${\tau }_{2u}=\frac{({{\varvec{n}}}_{2}^{*}{)}_{2}\cdot {\left({{\varvec{r}}}_{{2}{\text{u}}\psi }^{*}\right)}_{2}}{\sqrt{{\left[{\left({{\varvec{r}}}_{{2}{\text{u}}}^{*}\right)}_{2}\right]}^{2}{\left[{\left({{\varvec{r}}}_{{2}\psi }^{*}\right)}_{2}\right]}^{2}-{\left[{\left({{\varvec{r}}}_{{2}{\text{u}}}^{*}\right)}_{2}\cdot {\left({{\varvec{r}}}_{{2}\psi }^{*}\right)}_{2}\right]}^{2}}}=\frac{({{\varvec{n}}}_{2}^{*}{)}_{2}\cdot \frac{{\partial }^{2}({{\varvec{r}}}_{2}^{*}{)}_{2}}{\partial {\psi }_{2}\partial {u}_{2}}}{\sqrt{{\left[\frac{\partial ({{\varvec{r}}}_{2}^{*}{)}_{2}}{\partial {u}_{2}}\right]}^{2}{\left[\frac{\partial ({{\varvec{r}}}_{2}^{*}{)}_{2}}{\partial {\psi }_{2}}\right]}^{2}-{\left[{\left(\frac{\partial ({{\varvec{r}}}_{2}^{*}{)}_{2}}{\partial {u}_{2}}\right)}_{2}\cdot \frac{\partial ({{\varvec{r}}}_{2}^{*}{)}_{2}}{\partial {\psi }_{2}}\right]}^{2}}},$$

24 $${\tau }_{2\psi }=\frac{{\kappa }_{2\psi }\cdot {\left({{\varvec{r}}}_{{2}{\text{u}}}^{*}\right)}_{2}\cdot {\left({{\varvec{r}}}_{{2}\psi }^{*}\right)}_{2}}{\sqrt{{\left[{\left({{\varvec{r}}}_{{2}{\text{u}}}^{*}\right)}_{2}\right]}^{2}{\left[{\left({{\varvec{r}}}_{{2}\psi }^{*}\right)}_{2}\right]}^{2}-{\left[{\left({{\varvec{r}}}_{{2}{\text{u}}}^{*}\right)}_{2}\cdot {\left({{\varvec{r}}}_{{2}\psi }^{*}\right)}_{2}\right]}^{2}}}-{\tau }_{2u}$$

**Figure 5:**
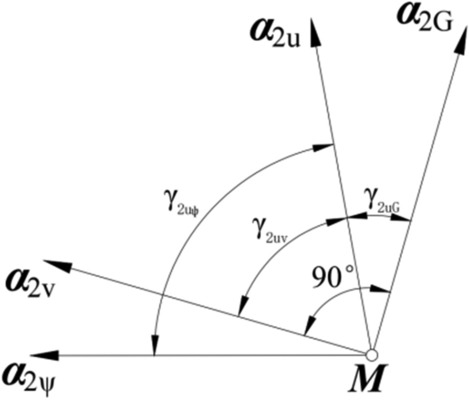
Directions of the tangent vectors to the parameter curves, tooth profile curve, and tooth trace curve at *M*.

As shown in Figs. 4 and 5, an initial position of the gear is the position in which the reference point *M*, located in the gear pitch cone generatrix. Angle *φ*_M_ of the gear rotation about its axis ***k***_2_ is measured from initial positions. Vector ***m***_2_(*φ*_M_, *δ*_2_) is the unit vector of the gear pitch cone generatrix and ***n***_2_(*φ*_M_, *δ*_2_) is the normal vector of the gear pitch cone surface at point *M* (Fig. 4). Parameter *α*_n2_ is the pressure angle of the gear tooth surface at point *M*. ***α***_2v_ is the unit vector for the direction of the tooth trace, and ***α***_2G_ is the unit vector for the direction of the tooth profile. ***α***_2v_ is perpendicular to ***α***_2G_. ***α***_2v_ and ***α***_2G_ are in the tangent planes to surface *Σ*_2_ at point *M*, and ***α***_2v_ can be represented in coordinate system *S*_2_ as:

25 $$({\boldsymbol{\alpha }}_{\text{2v}}{)}_{2}=\frac{{{\varvec{n}}}_{2}({\varphi }_{\text{M2}},{\delta }_{2})\times ({{\varvec{n}}}_{2}^{*}{)}_{2}}{\mathit{sin}(9{0}^{\circ }-{\alpha }_{n})},$$

26 $$({\boldsymbol{\alpha }}_{\text{2G}}{)}_{2}=({\boldsymbol{\alpha }}_{\text{2v}}{)}_{2}\times ({{\varvec{n}}}_{2}^{*}{)}_{2}.$$

Here,

27 $${{\varvec{n}}}_{2}({\varphi }_{M2},{\delta }_{2})=\left[\begin{array}{c}\mathit{cos}{\delta }_{2}\mathit{cos}{\varphi }_{M2}\\ \mathit{cos}{\delta }_{2}\mathit{sin}{\varphi }_{M2}\\ \begin{array}{c}-\mathit{sin}{\delta }_{2}\\ 1\end{array}\end{array}\right],$$

28 $${\varphi }_{\text{M2}}=\mathit{arctan}(\frac{{y}_{2}}{{x}_{2}}),$$

29 $${\alpha }_{n}=\mathit{arcsin}\left[{{\varvec{n}}}_{2}({\varphi }_{M2},{\delta }_{2})\cdot ({{\varvec{n}}}_{2}^{*}{)}_{2}\right].$$

The angle between vectors ***α***_2u_ and ***α***_2v_ is determined by

30 $${\gamma }_{2u\psi }=\mathit{arccos}\left[({\boldsymbol{\alpha }}_{\text{2u}}{)}_{2}\cdot ({\boldsymbol{\alpha }}_{\text{2v}}{)}_{2}\right].$$

The normal curvatures and the geodesic torsion for the directions of the tooth trace and the tooth profile can be represented respectively by.

31 $${\kappa }_{\text{2v}}=\left[{\kappa }_{{2}\psi }+({\tau }_{{2}\psi }-{\tau }_{\text{2u}})/{\mathit{tan}\gamma }_{{\text{2u}}\psi }\right]{{\mathit{sin}}^{2}\gamma }_{\text{2uv}}+{\tau }_{\text{2u}}\mathit{sin}2{\gamma }_{{\text{2u}}{\text{v}}}.$$

32 $${\kappa }_{\text{2G}}=\left[{\kappa }_{{2}\psi }+({\tau }_{{2}\psi }-{\tau }_{\text{2u}})/{\mathit{tan}\gamma }_{{\text{2u}}\psi }\right]{{\mathit{cos}}^{2}\gamma }_{\text{2uv}}-{\tau }_{\text{2u}}\mathit{sin}2{\gamma }_{\text{2uv}}.$$

33 $${\tau }_{\text{2v}}=0.5\left[{\kappa }_{{2}\psi }+({\tau }_{{2}\psi }-{\tau }_{\text{2u}})/{\mathit{tan}\gamma }_{{\text{2u}}\psi }\right]\mathit{sin}2{\gamma }_{\text{2uv}}+{\tau }_{\text{2u}}\mathit{cos}2{\gamma }_{\text{2uv}}.$$

34 $${\tau }_{\text{2G}}=-{\tau }_{\text{2v}}$$

The normal curvatures and the geodesic torsion of the generating gear tooth surface *Σ*_4_ of the pinion for the directions of the tooth trace and the tooth profile can be represented and denoted respectively by *κ*_4v_, *τ*_4v_, *κ*_4G_, *τ*_4G_, and *γ*_4uv_, *γ*_4u*ψ*_. The detailed derivations, fully provided in Reference 22, are the same as Eqs. (18–34).

In the generation method, the pinion tooth surface *Σ*_1_ is the envelope of the family of generating surfaces *Σ*_4_. Based on the meshing theory, the normal curvatures and the geodesic torsion (*κ*_1v_, *κ*_1G_, and *τ*_1v_) of surface *Σ*_1_ for tangent vectors ***α***_1v_ and ***α***_1G_ of the tooth trace and the tooth profile at point *M* are determined with respect to the curvature relations between the mating surfaces and represented by the following equations:

35 $${\kappa }_{\text{1v}}={\kappa }_{\text{4v}}-{\kappa }_{\text{41v}}.$$

36 $${\kappa }_{\text{1G}}={\kappa }_{\text{4G}}-{\kappa }_{\text{41G}}.$$

37 $${\tau }_{\text{1v}}={\tau }_{\text{4v}}-{\tau }_{\text{41v}}.$$

Here, *κ*_41v_, *κ*_41G_, and *τ*_41v_ are the induction normal curvatures and the induction geodesic torsion, respectively, and their detailed derivation is provided in Reference^22^.

### Direction angle of the contact path at the reference point

**Figure 6:**
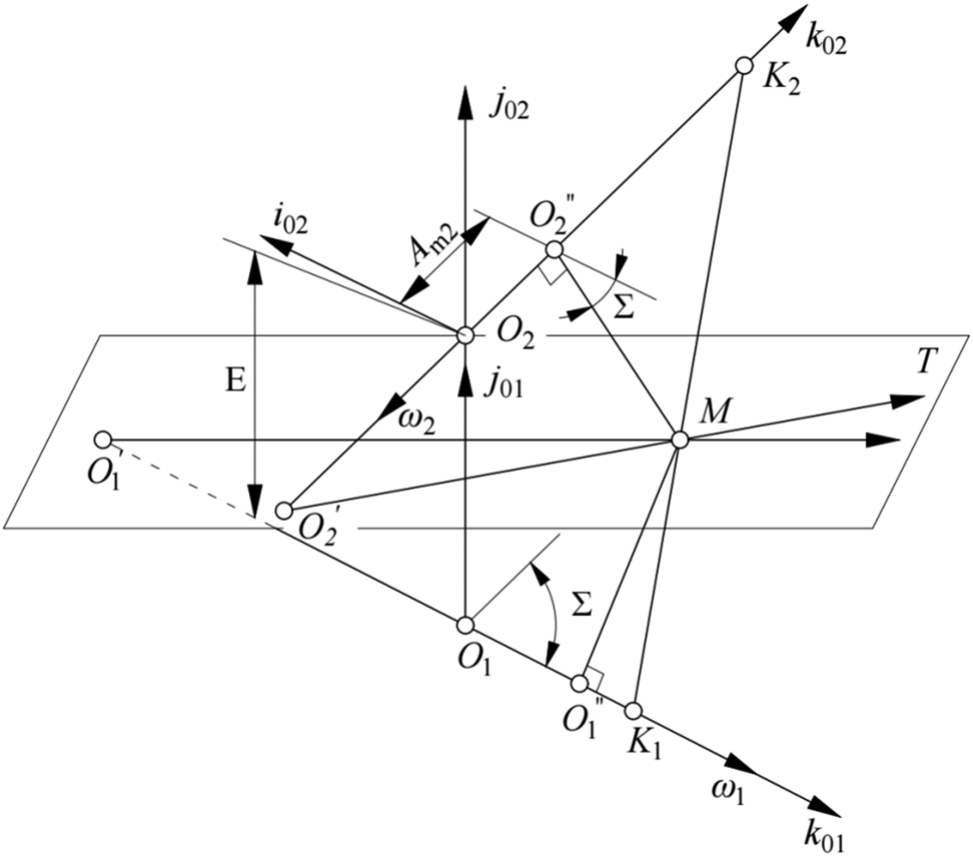
Coordinate systems connected to the machine frame.

Figure 6 shows the relative position of the pinion and gear when they are meshing at point *M*. Coordinate system *S*_0*i*_(*O*_*i*_; ***i***_0*i*_*, ****j***_0*i*_*, ****k***_0*i*_) (*i* = 1, 2) is rigidly connected to the machine frame. Origin *O*_*i*_ is the foot point of the common perpendicular of the axis of the pinion and the axis of the gear; axis ***k***_0*i*_ coincides with the axis of the blank, and axis ***j***_0*i*_ coincides with the common perpendicular. Origin *O*″_2_ is in the center of the pitch circle of the gear at point *M*. Parameter *E* is the offset, and parameter *Σ* is the shaft angle.

By coordinate transformation, the gear tooth surface *Σ*_2_ is represented in system *S*_02_ as (based on Fig. 6):

38 $$({{\varvec{r}}}_{2}{)}_{02}={A}_{\text{m2}}{{\varvec{k}}}_{02}+{{\varvec{M}}}_{202}\cdot ({{\varvec{r}}}_{2}^{*}{)}_{2},$$

Where

39 $${{\varvec{M}}}_{202}=\left[\begin{array}{ccc}\mathit{cos}\left(18{0}^{\circ }-{\varphi }_{M2}+{\varepsilon }_{2}\right)& -\mathit{sin}\left(18{0}^{\circ }-{\varphi }_{M2}+{\varepsilon }_{2}\right)& \begin{array}{cc}0& 0\end{array}\\ \mathit{sin}\left(18{0}^{\circ }-{\varphi }_{M2}+{\varepsilon }_{2}\right)& \mathit{cos}\left(18{0}^{\circ }-{\varphi }_{M2}+{\varepsilon }_{2}\right)& \begin{array}{cc}0& 0\end{array}\\ \begin{array}{c}0\\ 0\end{array}& \begin{array}{c}0\\ 0\end{array}& \begin{array}{c}\begin{array}{cc}1& 0\end{array}\\ \begin{array}{cc}0& 1\end{array}\end{array}\end{array}\right],$$

40 $${\varepsilon }_{2}=\mathit{arcsin}(\mathit{sin}{\beta }_{\text{m12}}\mathit{cos}{\delta }_{1}/\mathit{sin}\Sigma ).$$

*A*_m2_ is the installment distance of the gear, *β*_m12_ is the difference between the spiral angles of the pinion and that of the gear, and *δ*_1_ is the pitch angle of the pinion.

**Figure 7:**
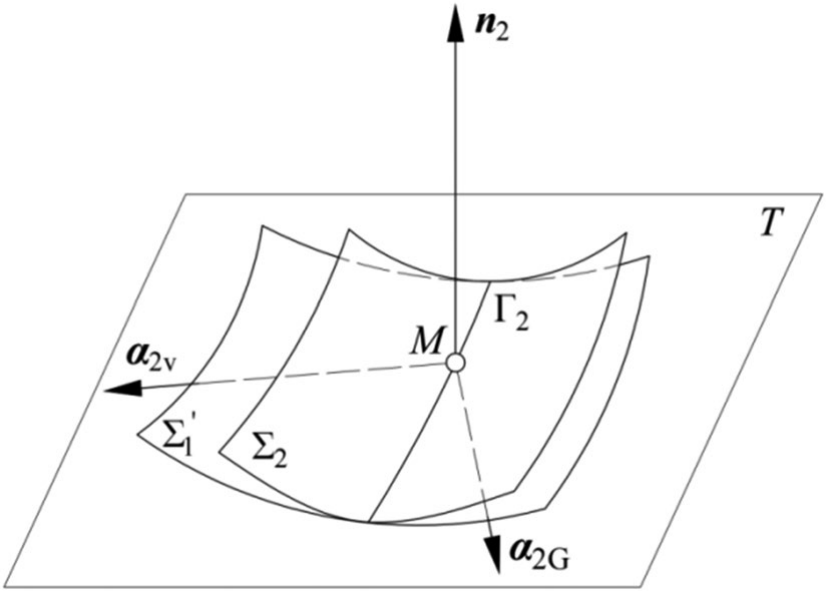
Relative positions of Σ_2_ and Σ_1_ and the coordinate system.

The vectors of the axes of coordinate system *S*_M_(*M*, ***α***_2v_, ***α***_2G_, ***n***_2_) are represented in system *S*_02_ as (based on Fig. 7):

41 $$({\boldsymbol{\alpha }}_{\text{2v}}{)}_{02}={{\varvec{M}}}_{202}\cdot ({\boldsymbol{\alpha }}_{\text{2v}}{)}_{2},$$

42 $$({\boldsymbol{\alpha }}_{\text{2G}}{)}_{02}={{\varvec{M}}}_{202}\cdot ({\boldsymbol{\alpha }}_{\text{2G}}{)}_{2},$$

43 $$({{\varvec{n}}}_{2}{)}_{02}={{\varvec{M}}}_{202}\cdot ({{\varvec{n}}}_{2}^{*}{)}_{2}.$$

The angle velocity of the pinion is defined as |*ω*_1_|= 1 rad·s^−1^, and the relative angular velocity is represented in system *S*_02_ by.

44 $$({{\varvec{\omega}}}_{12}{)}_{02}=({{\varvec{\omega}}}_{1}{)}_{02}-({{\varvec{\omega}}}_{2}{)}_{02},\boldsymbol{ }$$

45 $$({{\varvec{\omega}}}_{1}{)}_{02}=({{\varvec{k}}}_{01}{)}_{02}={{\varvec{M}}}_{21}\cdot {{\varvec{k}}}_{01},$$

46 $$({{\varvec{\omega}}}_{2}{)}_{02}=-\frac{1}{{i}_{12}}({{\varvec{k}}}_{02}{)}_{02},$$

where *i*_12_ is the transmission ratio at point *M*, and.

47 $${{\varvec{M}}}_{21}=\left[\begin{array}{cc}\begin{array}{c}\mathit{cos}\left(-\Sigma \right)\\ 0\\ \begin{array}{c}-\mathit{sin}\left(-\Sigma \right)\\ 0\end{array}\end{array}& \begin{array}{ccc}0& \mathit{sin}\left(-\Sigma \right)& 0\\ 1& 0& 0\\ \begin{array}{c}0\\ 0\end{array}& \begin{array}{c}\mathit{cos}\left(-\Sigma \right)\\ 0\end{array}& \begin{array}{c}0\\ 1\end{array}\end{array}\end{array}\right].$$

The relative velocity of the pinion and gear at point *M* is represented by.

48 $$({{\varvec{v}}}_{12}{)}_{02}=({{\varvec{\omega}}}_{12}{)}_{02}\times ({{\varvec{r}}}_{2}{)}_{02}+({{\varvec{\omega}}}_{1}{)}_{02}\times \left(E\cdot {{\varvec{j}}}_{02}\right).$$

In the meshing process, surfaces *Σ*_1_ and *Σ*_2_ are in point contact continuously, and it can be assumed that imaginary tooth surfaces *Σ*_2_′ and *Σ*_1_′ is obtained from the envelopes of surfaces *Σ*_1_ and *Σ*_2_, and they are in line contact respectively. ***α***_1v_ and ***α***_2v_ are the vectors for the directions of the tooth trace of the pinion and the gear. At point *M*, ***α***_1v_ = ***α***_2v_. Surfaces *Σ*_2_ and *Σ*_1_′, as shown in Fig. 7, are in line contact. Γ_2_ is the contact path. The relative positions of *Σ*_1_ and *Σ*_2_′are the same as those of *Σ*_2_ and *Σ*_1_′.

Tooth surface *Σ*_1_ is in conjugation with surface *Σ*_2_′; thus, the unit normal vector of the instantaneous contact line is obtained as.

49 $$({{\varvec{N}}}_{12}{)}_{02}={N}_{\text{12v}}\cdot ({\boldsymbol{\alpha }}_{\text{2v}}{)}_{02}+{N}_{12G}\cdot ({\boldsymbol{\alpha }}_{\text{2G}}{)}_{02},$$

Here,

50 $${N}_{\text{12v}}={\kappa }_{\text{1v}}({{\varvec{v}}}_{12}{)}_{02}\cdot ({\boldsymbol{\alpha }}_{\text{2v}}{)}_{02}+{\tau }_{\text{1v}}({{\varvec{v}}}_{12}{)}_{02}\cdot ({\boldsymbol{\alpha }}_{\text{2G}}{)}_{02}+({{\varvec{\omega}}}_{12}{)}_{02}\cdot ({\boldsymbol{\alpha }}_{\text{2G}}{)}_{02},$$

51 $${N}_{\text{12G}}={\tau }_{\text{1v}}({{\varvec{v}}}_{12}{)}_{02}\cdot ({\boldsymbol{\alpha }}_{\text{2v}}{)}_{02}+{\kappa }_{\text{1v}}({{\varvec{v}}}_{12}{)}_{02}\cdot ({\boldsymbol{\alpha }}_{\text{2G}}{)}_{02}-({{\varvec{\omega}}}_{12}{)}_{02}\cdot ({\boldsymbol{\alpha }}_{\text{2v}}{)}_{02}.$$

Tooth surface *Σ*_2_ is in conjugation with tooth surface *Σ*_1_′; thus, the unit normal vector of the instantaneous contact line is obtained as.

52 $$({{\varvec{N}}}_{21}{)}_{02}={N}_{\text{21v}}({\boldsymbol{\alpha }}_{\text{2v}}{)}_{02}+{N}_{\text{21G}}({\boldsymbol{\alpha }}_{\text{2G}}{)}_{02},$$

Here,

53 $${N}_{\text{21v}}=-{\kappa }_{\text{2v}}({{\varvec{v}}}_{12}{)}_{02}\cdot ({\boldsymbol{\alpha }}_{\text{2v}}{)}_{02}-{\tau }_{\text{2v}}({{\varvec{v}}}_{12}{)}_{02}\cdot ({\boldsymbol{\alpha }}_{\text{2G}}{)}_{02}-({{\varvec{\omega}}}_{12}{)}_{02}\cdot ({\boldsymbol{\alpha }}_{\text{2G}}{)}_{02},$$

54 $${N}_{\text{21G}}=-{\tau }_{\text{2v}}({{\varvec{v}}}_{12}{)}_{02}\cdot ({\boldsymbol{\alpha }}_{\text{2v}}{)}_{02}-{\kappa }_{\text{2v}}({{\varvec{v}}}_{12}{)}_{02}\cdot ({\boldsymbol{\alpha }}_{\text{2G}}{)}_{02}+({{\varvec{\omega}}}_{12}{)}_{02}\cdot ({\boldsymbol{\alpha }}_{\text{2v}}{)}_{02}.$$

**Figure 8:**
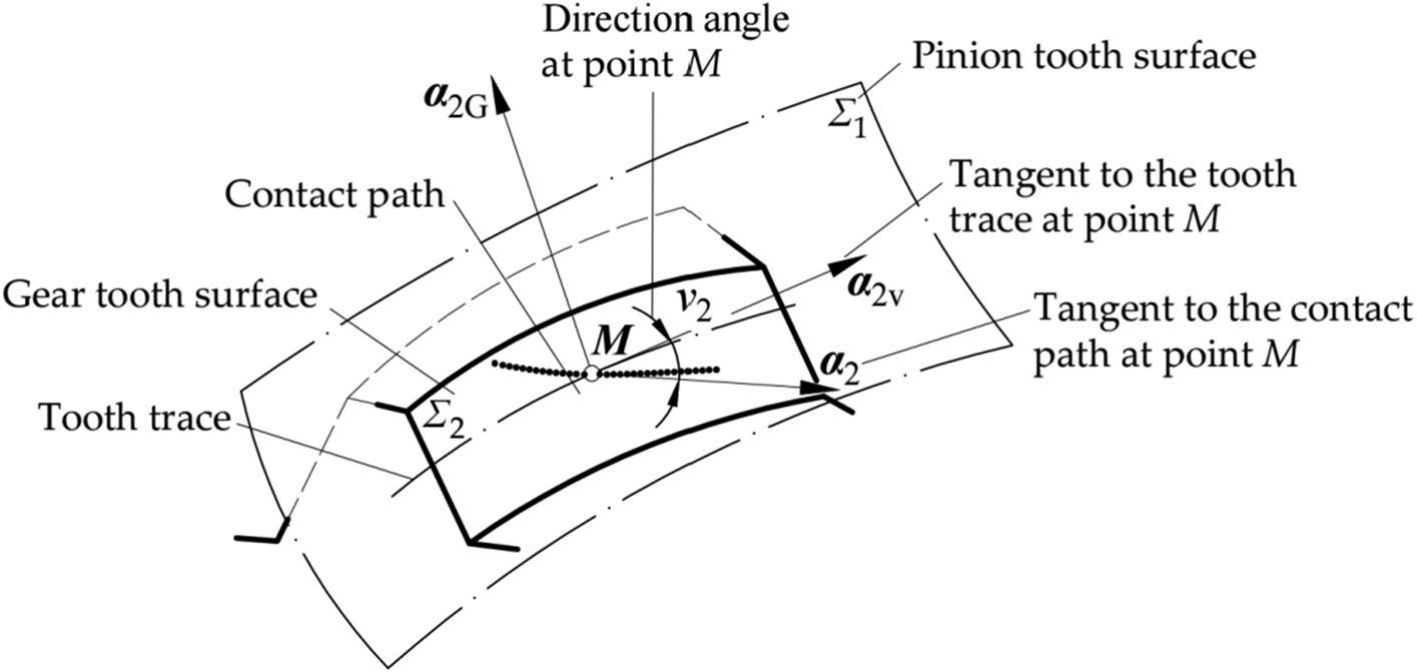
Position of the direction angle of the contact path.

The unit vectors tangent to the contact path at point *M* are denoted by ***α***_1_ and ***α***_2_ for the pinion and gear tooth surface, shown as Fig. 8, respectively. The direction angle of ***α***_*i*_ to ***α***_2v_ is defined as *ν*_*i*_ (*i* = 1, 2):

55 $${\boldsymbol{\alpha }}_{i}=\mathit{cos}{v}_{i}\cdot {\boldsymbol{\alpha }}_{\text{2V}}+\mathit{sin}{v}_{i}\cdot {\boldsymbol{\alpha }}_{\text{2G}}.$$

The area around reference point *M* meets the geometry condition of the tooth contact along the contact path: reference point *M* is the common point of both tooth surfaces, and a common normal line for both tooth surfaces exists at reference point *M*. Accordingly, angle *ν*_*i*_ is obtained by the following equations:

56 $${\mathit{tan}\nu }_{2}=\frac{-\left({\kappa }_{12V}+{\tau }_{12V}\right)\mathit{tan}{\theta }_{12}}{{\kappa }_{12G}\mathit{tan}{\theta }_{12}+{\tau }_{12V}},$$

57 $${\mathit{tan}\nu }_{1}=\frac{-\left({\kappa }_{12V}+{\tau }_{12V}\right)\mathit{tan}{\theta }_{21}}{{\kappa }_{12G}\mathit{tan}{\theta }_{21}+{\tau }_{12V}},$$

Where

58 $$\mathit{tan}{\theta }_{12}=\frac{{N}_{12V}}{{N}_{12G}},$$

59 $$\mathit{tan}{\theta }_{21}=\frac{{N}_{21V}}{{N}_{21G}}.$$

## Influence of the Angular Setting of the Head Cutter on the Direction of the Contact Path

In the Spirac system, the installment of the head cutter is determined by parameters *E*_x*i*_ and *q*_*i*_ for the gear if *i* = 2 and for the pinion if *i* = 1^22^. *E*_x*i*_ is corrected to satisfy the requirement of the spiral angle at the reference point, whereas *q*_*i*_ is not corrected. To further satisfy the requirement of the direction angle of the contact path, one more parameter is required. Thus, *q*_1_ is chosen, and its influence on the contact path is investigated.

A numerical example is considered to analyze the influence, and Table 1 shows the main design parameters of the gear and the pinion.

**Table 1:** Design parameters for the numerical example.

Items			Pinion		Gear
Number of teeth	*z*	(–)	10		41
Shaft angle	*Σ*	(°)		90	
Mean normal module	*m*_n_	(mm)		3.3342	
Offset	*E*	(mm)		20	
Face width	*b*	(mm)	33		30
Mean spiral angle	*β*_m_	(°)	50.01LH		37.1344RH
Pressure angle	*α*_n_	(°)		20	
Addendum coefficient	*h*^∗^	(–)		1	
Bottom clearance coefficient	*c*^∗^	(–)		0.25	
Addendum	*h*_a_	(mm)	4.67		2
Whole depth height	*h*	(mm)		7.5	
Reference cone angle	*δ*	(°)	17.614		71.9607
Number of cutter tarts	*z*_0_	(–)		11	
Cutter radius	*r*_0_	(mm)		74	
Tilt angle	*χ*	(°)	5		4

**Figure 9:**
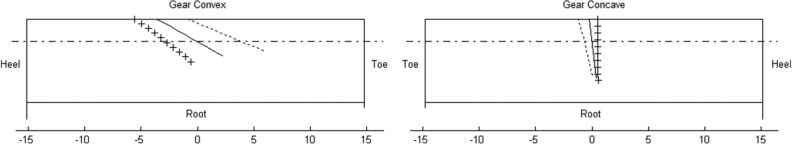
Influence of q4 on the contact path.

Figure 9 shows the result of the contact path on the gear tooth surfaces obtained by correcting the value of *q*_1_. The dotted lines with “ + ” signify the contact paths when △*q*_1_ =  + 0*.*0003 rad, the dotted lines with “ − ” signify the contact paths when △*q*_1_ =  − 0*.*0003 rad, and the solid lines signify the contact paths when the value is not corrected.

In Fig. 9, the positive correction of *q*_1_ increases the angles between the contact path and the tooth trace on the concave and convex tooth surfaces simultaneously, whereas the negative correction decreases the angles. Meanwhile, the positive correction makes the contact paths on the concave and convex tooth surfaces move to the tooth heel simultaneously, whereas the negative correction has the reverse effect.

The result proves the validity of the angular setting of the head cutter *q*_1_ to control the direction of the contact path. Considering its influence on the contact path, *q*_1_ is added to control the symmetry of the contact paths on the concave and convex tooth surfaces of the gear.

## Building equations

In the face hobbing method, the concave and convex tooth surfaces of the pinion or the gear are cut simultaneously under a single set of machine tool settings. Equations (1–60), described above, can be applied to evaluate the concave tooth surfaces of the pinion and the gear with the replacement of parameters *u*_1i_, *ψ*_1i_, and *α*_1i_, and *u*_2e_, *ψ*_2e_, and *α*_2e_ with the original parameters (*u*_1_, *ψ*_1_, and *α*_1_, and *u*_2_, *ψ*_2_, and *α*_2_) and the convex tooth surfaces with the replacement of parameters *u*_1e_, *ψ*_1e_, and *α*_1e_, and *u*_2i_, *ψ*_2i_, and *α*_2i_ with the original parameters (*u*_1_, *ψ*_1_, and *α*_1_, and *u*_2_, *ψ*_2_, and *α*_2_).

If the contact paths of the concave and convex tooth surfaces are symmetrical, parameters *ν*_1e_ and *ν*_1i_ will be related by the following equation:

60 $${\nu }_{\text{1e}}=-{\nu }_{\text{1i}}.$$

The current computation of machine tool settings for the Spirac hypoid gears is based on three conditions at the reference point: (a) the reference point is at the prescribed location, (b) the pressure angle at the reference point is equal to the theoretical value, and (c) the spiral angle at the reference point is equal to the theoretical value. The equations are.

62 $$\left\{\begin{array}{c}{r}_{mjk}^{r}={r}_{m2}\\ {r}_{mjk}^{n}=0\\ {\alpha }_{mjk}={\alpha }_{mk}\\ {\beta }_{mjk}={\beta }_{m2}\end{array}\right.\left(j=\text{4,2};k=i,e\right),$$

Where ${r}_{mjk}^{r}$ and ${r}_{mjk}^{n}$ are the radial and axial positions of the reference point, respectively, on tooth surface Σ_*j*_ in coordinate system *S*_*j*_; *r*_m2_ is the radius of the reference circle at point *M*; ***α***_m4i_ and ***α***_m4e_, and ***α***_m2e_ and ***α***_m2i_ are the normal pressure angles of the concave and convex tooth surfaces of the generating gear of pinion and the gear at the reference point, respectively; *α*_mi_ and *α*_me_ are the theoretical values of the normal pressure angles; *β*_m4i_ and *β*_m4e_, and *β*_m2e_ and *β*_m2i_ are the spiral angles of the concave and convex tooth surfaces of the generating gear of pinion and the gear at the reference point, respectively; *β*_m2_ is the theoretical value of the spiral angle at the reference point.

Equation (61) consists of 16 nonlinear equations, which contain 16 unknowns, *u*_1*k*_, *ψ*_1*k*_, *u*_2*k*_, *ψ*_2*k*_, *E*_x1_, *E*_x2_, *α*_1*k*_,* α*_2*k*_, *δ*_40_, and *δ*_M2_, for solving for a set of values. To achieve symmetry of the contact paths, angle *q*_1_ is taken as an unknown, and Eq. (60) is used for another function. Overall, 17 equations obtained from Eqs. (60) and (61) are applied to solve for the 17 unknowns to achieve the final values of the machine tool settings.

## Analysis of the symmetry of the contact path

**Figure 10:**
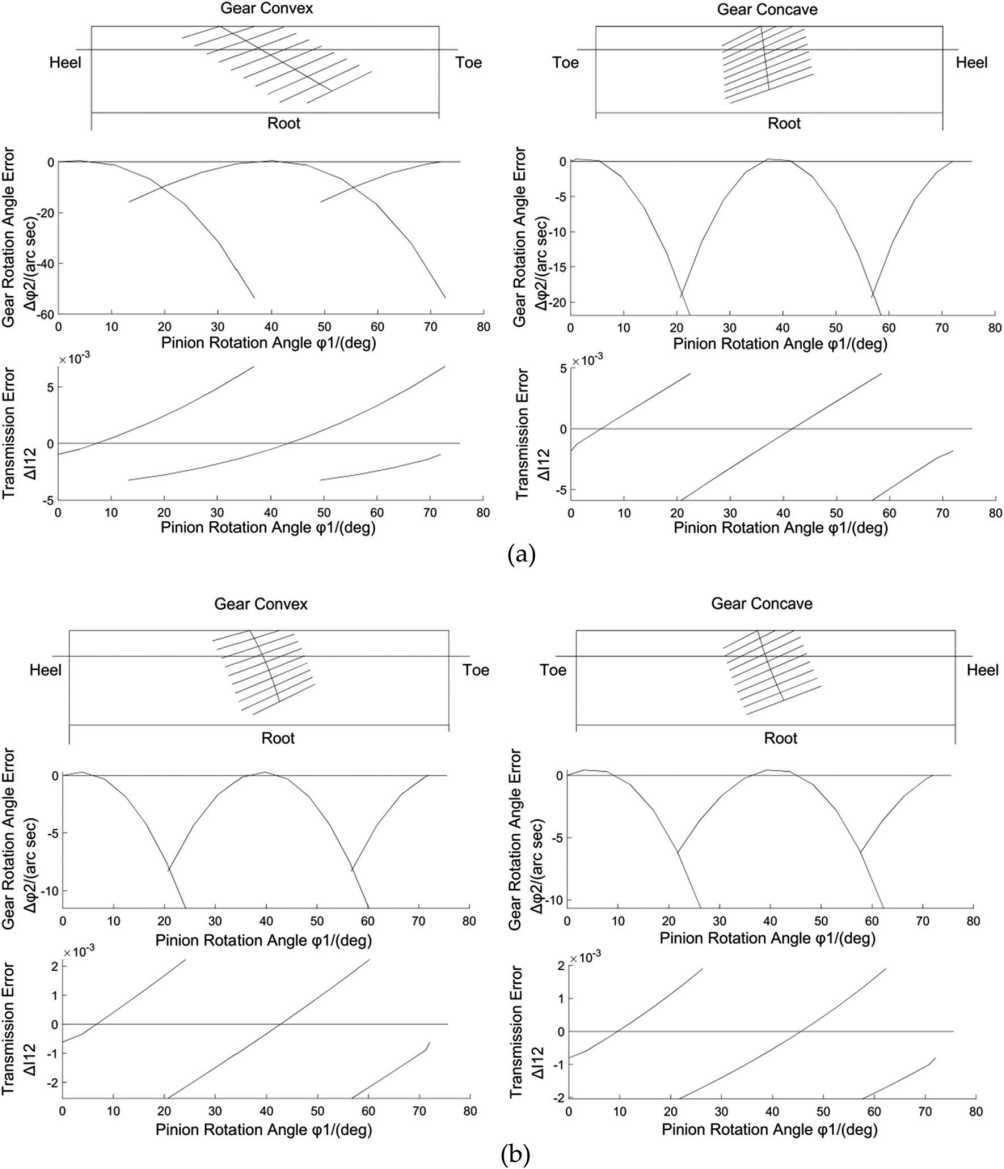
TCA outputs with the (**a**) original and (**b**) new methods.

Table 1 lists the design parameters for the example hypoid gear pair. By the original method which is currently used for calculation^22^, the values of the machine tool settings are obtained and listed in Tables 2, and 3 lists the results of the conditions. The output of the tooth contact analysis (TCA) is shown in Fig. 10a.

**Table 2:** Machine tool settings by the original method for the numerical example.

Items		Concave		Convex
1) Data of the gear				
Distance parameter of the blade	*u*_2_(mm)	− 1.4909		− 1.3537
Angle parameter of the head cutter	*ψ*_2_(°)	0.0198		− 0.1392
Radial setting of the head cutter	*E*_x2_(mm)		98.6396	
Blade angle	*α*_2_(°)	− 26.0412		13.8861
Angle of installment	*δ*_M2_(°)		72.0903	
2) Data of the pinion				
Distance parameter of the blade	*u*_1_(mm)	1.4705		1.3759
Angle parameter of the head cutter	*ψ*_1_(°)	0.1771		− 0.0454
Radial setting of the head cutter	*E*_x1_(mm)		98.7660	
Blade angle	*α*_1_(°)	− 22.5719		17.3196
Pitch angle of the generating gear	*δ*_4_(°)		68.8477	
Angular setting of the head cutter	*q*_1_(°)		55.5783

**Table 3:** Results obtained by the original method for the numerical example.

Items		Concave		Convex
1) Data of the gear				
Radial position of the reference point	*r*^r^_m2_(mm)	85.7373		85.7373
Axial positions of the reference point	*r*^n^_m2_(°)	0		0
Spiral angle at the reference point	*β*_m2_(mm)	37.1344		37.1344
Pressure angle at the reference point	*α*_n2_(°)	22.1180		17.8820
2) Data of the generating gear of the pinion				
Radial position of the reference point	*r*^r^_m4_(mm)	85.7373		85.7373
Axial positions of the reference point	*r*^n^_m4_(°)	0		0
Spiral angle at the reference point	*β*_m4_(mm)	37.1344		37.1344
Pressure angle at the reference point	*α*_n4_(°)	17.8820		22.1180
Direction angle of the contact paths	*ν*_1_(°)	16.0206		− 38.1097

By the new method proposed in this paper, the values of the machine tool settings are obtained and listed in Tables 4, and 5 lists the results obtained under the different conditions. The outputs of the TCA are shown in Fig. 10b.

**Table 4:** Machine tool settings obtained by the proposed method.

Items		Concave		Convex
*1) Data of the gear*				
Distance parameter of the blade	*u*_2_(mm)	− 1.4909		− 1.3537
Angle parameter of the head cutter	*ψ*_2_(°)	0.0198		− 0.1392
Radial setting of the head cutter	*E*_x2_(mm)		98.6396	
Blade angle	*α*_2_(°)	− 26.0412		13.8861
Angle of installment	*δ*_M2_(°)		72.0903	
2) Data of the pinion				
Distance parameter of the blade	*u*_1_(mm)	0.9016		0.6749
Angle parameter of the head cutter	*ψ*_1_(°)	− 9.4321		− 9.8387
Radial setting of the head cutter	*E*_x1_(mm)		98.8282	
Blade angle	*α*_1_(°)	− 26.5512		13.2689
Pitch angle of the generating gear	*δ*_4_(°)		71.4439	
Angular setting of the head cutter	*q*_1_(°)		65.2102

**Table5:** Results obtained by the proposed method.

Items		Concave		Convex
1 Data of the gear				
Radial position of the reference point	*r*^r^_m2_(mm)	85.7373		85.7373
Axial positions of the reference point	*r*^n^_m2_(°)	0		0
Spiral angle at the reference point	*β*_m2_(mm)	37.1344		37.1344
Pressure angle at the reference point	*α*_n2_(°)	22.1180		17.8820
2 Data of the generating gear of the pinion				
Radial position of the reference point	*r*^r^_m4_(mm)	85.7373		85.7373
Axial positions of the reference point	*r*^n^_m4_(°)	0		0
Spiral angle at the reference point	*β*_m4_(mm)	37.1344		37.1344
Pressure angle at the reference point	*α*_n4_(°)	17.8820		22.1180
Direction angle of the contact paths	*ν*_1_(°)	31.8615		− 31.8615

Tables 2 and 4 show that the machine tool settings for the gear are modified and determined to be equal, whereas the machine tool settings for the pinion change. Tables 3 and 5 show that the values obtained by the two methods, related to the position of the reference point, the pressure angle, and the spiral angle, are both equal to the theoretical values. Table 3 shows that the direction angle of the contact paths on the concave tooth surface is not equal to the angle on the convex by the original method. In order to obtain a good meshing performance, they need to be modified artificially and repeatedly by correcting the machine tool settings. However, the direction angles of the contact paths on the concave and convex tooth surfaces of the gear obtained by the proposed method are equal in Table 5. This method is practicable and effective for impacting the meshing performance.

In Fig. 10a, there are significant differences in the tooth contact quality and the motion error between the concave and convex tooth surfaces: (i) both contact paths are not symmetrical; (ii) the convex tooth surface of the gear has a small rotation angle error but a long contact zone, which increases the gear sensitivity; and (iii) although the concave tooth surface has a good contact zone, it has a large rotation angle error.

In Fig. 10b, the contact paths on the concave and convex tooth surfaces are approximately symmetrical, the motion errors are approximately equal, and the meshing characteristics are approximate. Thus, the proposed method for controlling the direction angle of the contact path at the reference point is effective, and it can improve the tooth contact quality and the transmission performance.

## Conclusion

In this paper, a novel optimization method is presented for determining machine tool settings specific to Spirac hypoid gears, aiming to achieve optimal meshing performance with greater efficiency.

In the existing Spirac system, the pinion is cut by the generated method, the gear is cut by the non-generated method, which provides a higher production, but the meshing performance cannot be predicted and controlled. It needs to be modified artificially and repeatedly by correcting the machine tool settings during the tooth contact analysis. In the proposed method, the symmetry of the contact paths on the concave and convex tooth surfaces of the gear is controlled to achieve optimal meshing performance with greater efficiency. The direction angles of the two contact paths are equated to estimate their symmetry, achieved by modifying the angular setting of the head cutter when cutting the pinion. The results show that the contact paths on the concave and convex tooth surfaces are approximately symmetrical and the transmission errors of both sides are comparable.

## Data Availability

All data generated or analyzed during this study are included in this published article.
